# The Effect of Thermocycling on the Microhardness of Contemporary Glass Ionomer-Based Restorative Materials: An In Vitro Study

**DOI:** 10.3390/bioengineering13020161

**Published:** 2026-01-29

**Authors:** Enes Bardakci, Didem Ozdemir Ozenen, Izzet Yavuz

**Affiliations:** 1 Department of Pediatric Dentistry, Harran University Faculty of Dentistry, Sanliurfa 63050, Turkey; 2 Arthur A. Dugoni School of Dentistry, University of the Pacific, San Francisco, CA 94103, USA; 3 Department of Pediatric Dentistry, Dicle University Faculty of Dentistry, Diyarbakır 21280, Turkey

**Keywords:** dental materials, glass ionomer cements, thermocycling, surface microhardness, pediatric dentistry

## Abstract

Glass ionomer-based restorative materials are widely used in pediatric dentistry because of their chemical adhesion to tooth structure, ion-releasing capacity, and clinical handling advantages; however, their mechanical durability under simulated oral aging conditions remains a critical factor influencing long-term clinical performance. This in vitro study aimed to evaluate and compare the surface microhardness of three contemporary glass ionomer-based restorative materials—Beautifil Bulk Restorative, EQUIA Forte HT, and Fuji II LC—before and after thermocycling. A total of 90 disc-shaped specimens (10 mm in diameter and 2 mm in thickness) were prepared, with 30 samples allocated to each material group. Microhardness measurements were performed using the Vickers hardness test at baseline and after 10,000 thermocycling cycles between 5 °C and 55 °C to simulate intraoral aging. Results were expressed as the mean ± standard deviation, and statistical analyses were conducted using non-parametric tests. Thermocycling resulted in a statistically significant reduction in microhardness values for all tested materials (*p* < 0.05). Beautifil Bulk Restorative exhibited the highest microhardness values both before and after thermocycling, followed by Fuji II LC and EQUIA Forte HT, with significant differences observed among all groups (*p* < 0.001). Within the limitations of this study, Beautifil Bulk Restorative may be considered a favorable option for restorations in young permanent teeth, whereas EQUIA Forte HT, exhibiting lower microhardness values, may be more suitable for primary teeth, where physiological wear is expected.

## 1. Introduction

Dental caries is one of the most prevalent chronic diseases of childhood and remains a major public health concern due to its multifactorial and infectious nature. When carious lesions progress beyond the point of remineralization, restorative intervention becomes necessary. In pediatric restorative dentistry, the selection of an appropriate restorative material is critical and must consider several factors, including adhesion to dental tissues, resistance to masticatory forces, biocompatibility, and ease of clinical application. Within this context, glass ionomer-based materials have gained prominence as versatile systems that fulfill many of these clinical requirements [1,2,3].

Glass ionomer cements (GICs) were first introduced by Wilson and Kent in 1972 for restorative and protective purposes. They offer several advantages, including chemical adhesion to tooth structure, tolerance to moisture, absence of heat generation during setting, anticariogenic effects, antibacterial activity, biocompatibility, and a thermal expansion coefficient similar to that of natural tooth tissue [4,5]. However, their relatively low mechanical strength—particularly surface microhardness—continues to limit their long-term clinical durability. Although resin-modified GICs (RMGICs) and high-viscosity GICs (HVGICs) were developed to address these shortcomings, their mechanical behavior following intraoral aging remains a subject of concern [6]. Giomer-based bulk-fill composite materials represent a more recent class of restorative systems that combine the esthetic and mechanical advantages of resin composites with the bioactive features of surface pre-reacted glass (S-PRG) fillers. Despite their increasing use, comparative evidence evaluating their resistance to aging and mechanical degradation relative to contemporary GIC-based materials is still limited [7,8]. This knowledge gap highlights the need for systematic evaluation of these materials under conditions that simulate the dynamic oral environment, which is characterized by humidity, thermal fluctuations, chewing forces, and pH changes. Among various mechanical properties, surface microhardness is particularly important because it correlates with wear resistance, structural integrity, and overall restorative longevity [9,10].

From a biomimetic standpoint, restorative materials are expected to replicate not only the structural integrity of natural dental tissues but also their functional behavior under physiological conditions. Glass ionomer-based materials and giomers are considered biomimetic restorative systems due to their chemical adhesion to tooth structure, thermal expansion coefficients comparable to enamel and dentin, ion-releasing capacity that mimics natural remineralization processes, and controlled wear behavior under functional loading [3,11]. Materials that exhibit balanced mechanical stability while maintaining bioactivity may therefore better emulate the dynamic nature of dental tissues in the oral environment. In this context, evaluating the microhardness response of contemporary bioactive restorative materials under thermocycling is essential to assess their biomimetic performance under simulated intraoral aging conditions [4,5]. The aim of this study was to compare the microhardness values of an RMGIC, an HVGIC, and a packable bulk-fill composite material before and after thermocycling. By evaluating their performance under simulated aging conditions, the study seeks to provide clinically relevant information on the suitability of high-viscosity and bioactive restorative materials for pediatric dental applications. The null hypothesis (H_0_) was that thermocycling would not produce significant changes in the microhardness values of any of the tested restorative materials.

## 2. Material and Method

### 2.1. Study Design and Ethical Considerations

This in vitro study evaluated the microhardness of three contemporary restorative materials under simulated oral aging conditions. As no human participants or animals were involved, ethical committee approval was not required. The study was designed with three main groups and six independent subgroups to allow evaluation before and after thermocycling. For each material, 30 disc-shaped specimens were prepared, with 15 specimens allocated to baseline microhardness testing and 15 specimens allocated to thermocycling and subsequent microhardness evaluation. A priori sample size calculation was performed using the G*Power software (version 3.1.9.2), based on mean microhardness values and standard deviations reported in previous studies evaluating glass ionomer-based restorative materials [12,13]. Considering an effect size derived from these data, a significance level of α = 0.05, and a statistical power of 80% (1 − β = 0.80), the minimum required sample size was calculated as 30 specimens per material group. Accordingly, a total of 90 specimens were included in the study.

Specimens allocated to the thermocycling groups were randomly assigned using a computer-generated randomization list prior to the aging procedure.

### 2.2. Materials

Three commercially available restorative materials were selected based on their clinical relevance in pediatric dentistry (Table 1).

### 2.3. Specimen Preparation

Grouping:

• Group 1: Beautifil Bulk Restorative;
  ○ 1a: before thermocycling.
  ○ 1b: after thermocycling.
• Group 2: EQUIA Forte HT;
  ○ 2a: before thermocycling.
  ○ 2b: after thermocycling.
• Group 3: Fuji II LC;
  ○ 3a: before thermocycling.
  ○ 3b: after thermocycling.

**Figure 1:**
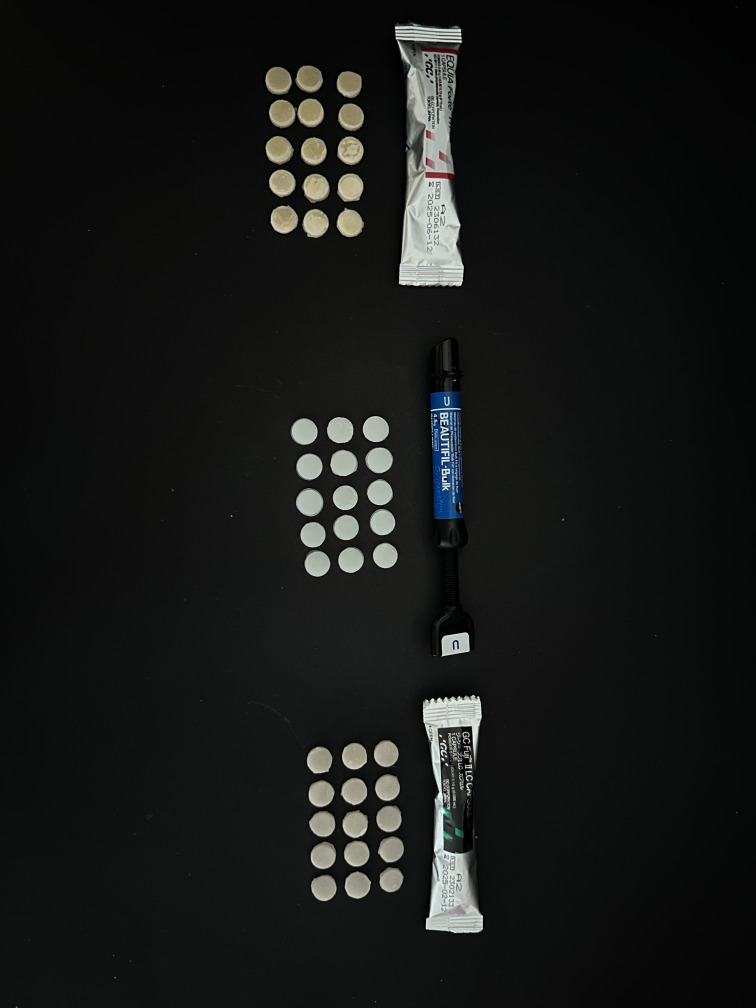
Image of disc-shaped specimens prepared for microhardness evaluation, illustrating the standardized dimensions (10 mm diameter, 2 mm thickness) applied to all groups.

Specimen fabrication:

• Teflon molds (diameter: 10 mm, height: 2 mm) were used to prepare 30 specimens per group. To minimize potential batch effects, all specimens were fabricated by the same operator, using identical molds, materials from the same batch numbers, and the same light-curing unit, within a single preparation period.
• The setting and curing times for each restorative material were selected in strict accordance with the manufacturers’ instructions for a 2-mm material thickness to ensure complete setting and optimal polymerization. Since surface microhardness is highly dependent on the degree of polymerization, curing time and light intensity were carefully standardized for all specimens to allow reliable inter-material comparison.
• Beautifil Bulk Restorative: The material was applied using a mouth spatula and light-cured with an LED unit (Bluephase G4, Ivoclar Vivadent, Liechtenstein) at 1500 mW/cm^2^ for 10 s per surface.
• EQUIA Forte HT and Fuji II LC: They were mixed in an amalgamator (Rock-mix, Dentmark, China) for 10 s, placed in molds, and light-cured for 20 s per surface using LED.
• The output irradiance of the LED curing unit was verified using a calibrated radiometer prior to specimen preparation to ensure consistent and accurate light intensity throughout the study.
• A transparent strip (Universal strips, Extra Dental, Istanbul, Türkiye) and a glass slide were placed over the molds during setting to obtain smooth and standardized surfaces. After setting, all specimens were polished using rubber polishing discs (Astropol, Ivoclar Vivadent, Schaan, Liechtenstein) in a sequential order (gray, green, and pink) for 30 s per surface under water cooling at approximately 10,000 rpm. Following polishing, specimens were stored in distilled water at 37 °C for 24 h prior to microhardness testing (Figure 1).

### 2.4. Thermocycling Procedure

After finishing and polishing, all specimens were randomly divided into two independent subgroups (n = 15 per subgroup). Specimens in the first subgroup served as the non-aged control and were subjected to microhardness testing without thermocycling, while specimens in the second subgroup were used exclusively for artificial aging and subsequent microhardness evaluation. Thus, different discs were used for pre-aging and post-aging measurements.

Thermocycling was performed for 10,000 cycles between 5 °C and 55 °C using a thermocycling device (model and manufacturer, if available). The specimens were alternately immersed in water baths maintained at the specified temperatures. Each cycle consisted of a dwell time of 30 s in each bath, with a transfer time of 10 s between baths. Distilled water was used as the cycling medium throughout the procedure.

The selected number of 10,000 cycles was chosen based on previous in vitro studies, where this protocol is commonly considered to simulate approximately one year of clinical service, reflecting thermal stresses encountered in the oral environment due to intake of hot and cold substances [14,15]. This approach allowed standardized artificial aging and enabled comparison with similar studies in the literature.

### 2.5. Microhardness Evaluation with Vickers Method

The microhardness of the samples was measured using the microhardness tester (Buehler Ltd., Lake Bluff, IL, USA) at the Yeditepe University Hard Tissue Laboratory.

For the microhardness measurement of restorative materials, a 200 g load was applied for 15 s and a trace was created with a square-based pyramid-shaped tip with a peak angle of 136 degrees. The resulting indentations were transferred from the microscope to the measurement screen via the computer connected to the device and subsequently measured. Microhardness measurements were performed on 15 specimens per subgroup. Baseline microhardness values were obtained from Groups 1a, 2a, and 3a, while post-thermocycling measurements were obtained from Groups 1b, 2b, and 3b. Each specimen was measured at three different points, and the mean of these three measurements was recorded as the Vickers microhardness value for that specimen.

### 2.6. Statistical Analysis

The data obtained in the study were analyzed using SPSS Statistics for Windows (version 25.0; IBM Corp., Armonk, NY, USA). Descriptive statistical methods (mean, standard deviation, median, minimum, and maximum values) were used to summarize the data. Normality of data distribution was assessed using the Shapiro–Wilk test, which indicated that the data were not normally distributed (*p* < 0.05). Therefore, non-parametric statistical tests were applied. Intragroup comparisons of surface microhardness values before and after thermocycling were performed using the Wilcoxon signed-rank test.

Intergroup comparisons among the three restorative materials were conducted using the Kruskal–Wallis H test, followed by Mann–Whitney U tests with Bonferroni correction for pairwise comparisons when statistically significant differences were detected. *p* values were reported as exact values or as *p* < 0.001, and a *p* value of < 0.05 was considered statistically significant.

## 3. Results

The Vickers microhardness values of the restorative materials before and after thermal cycling are presented in Table 2. The microhardness values of the prepared discs before thermal cycling revealed a significant difference between the materials (*p* < 0.05). As a result of the analysis, significant differences were observed between Groups 1a and 2a, between Groups 1a–3a and between Groups 2a–3a (*p* < 0.05). Accordingly, the microhardness value of Beautifil Bulk Restorative disc before thermal cycling is higher than EQUIA Forte HT and Fuji II LC. Also, the microhardness value of Fuji II LC is higher than EQUIA Forte HT.

Comparing the values obtained after thermal cycling, it was determined that there was a statistically significant difference between Group 1b, Group 2b, Group 3b (*p* < 0.001). Accordingly, the microhardness values of Beautifil Bulk Restorative discs after thermal cycling were higher than EQUIA Forte HT, Fuji II LC values; Fuji II LC values were higher than EQUIA Forte HT values (*p* < 0.001). In this case, it is seen that there is a high level of difference between the brands.

**Figure 2:**
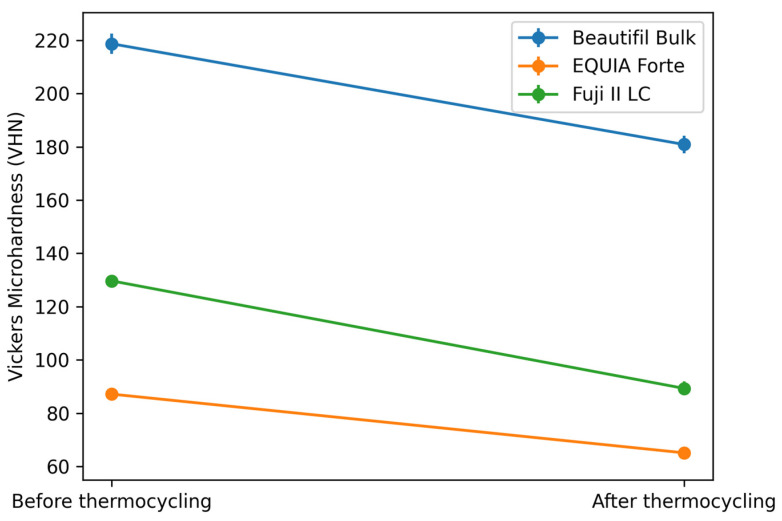
Comparison of Vickers microhardness (VHN) values of the tested restorative materials before and after thermocycling. Data are presented as the mean ± standard deviation.

It is seen at Figure 2 that a statistically significant difference was observed between the VHNs of the Beautifil Bulk Restorative disc before and after thermal cycling (*p* < 0.05).

A statistically significant difference was observed between VHNs of the Fuji II LC disc before thermal cycling and after thermal cycling (*p* < 0.05).

A statistically significant difference was observed between VHNs of the EQUIA Forte HT disc before thermal cycling and after thermal cycling (*p* < 0.05). The fact that the difference scores were in favor of negative ranks indicates that the microhardness values of the materials used in the study decreased.

## 4. Discussion

The present study evaluated the surface microhardness of three bioactive restorative materials—Beautifil Bulk Restorative, EQUIA Forte HT, and Fuji II LC—before and after thermocycling in order to simulate intraoral thermal aging conditions. The novelty of this study lies in the direct comparative evaluation of giomer-based, glass hybrid, and resin-modified glass ionomer restorative materials under standardized thermocycling conditions, providing clinically relevant insight into their mechanical stability in pediatric restorative dentistry. Unlike previous studies, which have largely focused on individual material categories, the present investigation offers a side-by-side comparison of different bioactive material systems under identical aging conditions, enabling a more comprehensive and clinically meaningful assessment of material performance. Significant differences in microhardness values were observed among all materials both prior to and following thermal cycling, indicating that material composition plays a critical role in mechanical durability. Accordingly, the null hypothesis (H_0_), which proposed that thermocycling would not induce significant changes in the microhardness of the tested materials, was rejected. Thermocycling resulted in a statistically significant reduction in microhardness across all material groups, demonstrating that simulated thermal aging adversely affects their mechanical stability.

Recently developed resin-based restorative materials, including bulk-fill composites and giomers, have gained increasing attention due to their enhanced mechanical performance, aesthetic qualities, and incorporation of bioactive filler technologies [16,17]. Beautifil Bulk Restorative, a giomer-based material evaluated in the present study, contains surface pre-reacted glass ionomer (S-PRG) fillers embedded within a resin matrix, which are known to contribute to fluoride release and acid-neutralising capacity. Prior to thermal cycling, Beautifil Bulk Restorative exhibited significantly higher Vickers microhardness values than both EQUIA Forte HT and Fuji II LC, while Fuji II LC demonstrated higher hardness than EQUIA Forte HT. These findings may be attributed to the hybrid resin–filler structure of giomers, which enhances filler–matrix bonding, limits water sorption, and improves resistance to mechanical degradation. Similar outcomes have been reported in previous studies, where bulk-fill giomer materials demonstrated superior microhardness compared with conventional glass ionomer and glass hybrid systems [18,19,20].

Fuji II LC, a RMGIC, exhibited intermediate microhardness values. Its resin-modified matrix may promote improved polymer cross-linking and reduced early water sensitivity, which could account for its higher hardness compared with EQUIA Forte HT. This observation is consistent with earlier studies reporting enhanced mechanical performance of RMGICs under aging conditions when compared with conventional or glass hybrid materials [21,22,23].

EQUIA Forte HT demonstrated the lowest microhardness values both before and after thermal cycling. Although this material was developed to address the mechanical limitations of conventional glass ionomer cements through modified glass technology and the application of a protective resin coating, its lower hardness may be associated with its glass-dominated matrix and higher ion-releasing potential [24]. While ion release is advantageous for remineralisation, it may also increase susceptibility to surface softening and mechanical degradation, particularly under thermal stress.

Thermal cycling resulted in a significant reduction in the microhardness of all tested materials, indicating that simulated intraoral aging negatively affects mechanical performance. The observed decrease following 10,000 thermal cycles may be explained by repetitive expansion–contraction stresses, water diffusion into the material matrix, and disruption of filler–matrix interfaces. These findings are consistent with previous reports demonstrating that thermocycling accelerates hydrolytic degradation and reduces surface hardness in bioactive restorative materials [13].

Surface hardness is a critical determinant of the clinical longevity of restorative materials, as it is directly related to resistance to wear, deformation, and surface damage under masticatory forces [25]. Materials with lower surface hardness are more susceptible to scratching and fatigue-related deterioration, which may compromise restoration longevity [26]. In line with established methodological recommendations, Vickers microhardness testing was employed in this study, as it is considered a reliable method for evaluating the hardness of dental restorative materials [13,27]. Despite the overall reduction in microhardness values, Beautifil Bulk Restorative maintained the highest hardness after thermal cycling, followed by Fuji II LC and EQUIA Forte HT. This consistent ranking suggests that the resin-based giomer structure offers greater resistance to thermal aging compared with glass hybrid and resin-modified glass ionomer systems. These findings are in agreement with the broader literature reporting material-dependent variations in the mechanical behavior of bioactive restorative systems.

In addition to the materials evaluated in the present study, several other bioactive restorative materials have been investigated in the literature with respect to their mechanical and biological behavior. Bioactive resin-based materials such as ACTIVA™ Kids have been reported to exhibit ion-releasing capability combined with resin matrix reinforcement, providing favorable biological properties while maintaining acceptable mechanical performance. However, previous studies have demonstrated that ACTIVA-based materials may present lower surface microhardness compared with conventional resin composites and giomer-based systems, particularly after aging procedures, which may limit their wear resistance under high functional load conditions [28,29].

Similarly, Ionolux, a light-cured, fluoride-releasing restorative material, has been shown to offer improved handling characteristics and ion release; however, its mechanical properties, including surface hardness, have been reported to be inferior to resin-based bioactive materials, especially following thermocycling and water storage [30]. Glass hybrid restorative systems such as Riva have been developed to enhance the strength and wear resistance of conventional glass ionomer cements through optimized glass fillers and resin coatings. Despite these improvements, studies indicate that glass hybrid materials still exhibit lower surface microhardness values compared with giomer and bulk-fill resin-based materials, particularly after simulated aging [31,32].

From a clinical perspective, surface microhardness is a key determinant of wear resistance, marginal integrity, and long-term restorative success. Based on the results of the present study, the tested restorative materials exhibited distinct microhardness profiles before and after thermocycling, which may directly influence their clinical indications. Materials demonstrating higher surface microhardness, such as Beautifil Bulk Restorative, may offer superior resistance to wear and mechanical degradation, making them particularly suitable for restorations in young permanent teeth exposed to higher functional loads. In contrast, materials with comparatively lower microhardness values, such as EQUIA Forte HT, may be more prone to surface abrasion under functional loading; however, they may still represent an appropriate choice for primary tooth restorations, where physiological wear is expected and excessive hardness may not be desirable. Fuji II LC exhibited an intermediate microhardness behavior, suggesting its suitability for clinical situations requiring a balance between mechanical durability and favorable handling properties. Overall, these findings suggest that restorative material selection should not rely solely on standardized classifications or ISO-based properties, but should also account for the specific clinical scenario, tooth type, and functional demands, thereby supporting a more individualized and evidence-based approach to restorative decision-making in pediatric dentistry.

### Study Limitations

This study has certain limitations. The experimental procedures and analyses were conducted at different locations, which may have introduced minor variability. In addition, sample preparation was performed under ideal in vitro conditions that do not fully reflect the challenges encountered in clinical practice. The use of thermocycling as an artificial ageing method cannot completely replicate the complex oral environment, including saliva, pH fluctuations, masticatory forces and tooth brushing. Furthermore, although numerous restorative materials are available on the market, only three materials were evaluated in this study. Another limitation of the present study is that surface microhardness was the only outcome measure evaluated. Other properties related to bioactivity and overall biomimetic performance, such as ion release, remineralization potential, wear resistance, or flexural strength, were not assessed and should be investigated in future studies. From a clinical perspective, the observed differences in surface microhardness after thermal cycling may have implications for restoration durability, particularly in pediatric patients exposed to frequent thermal and mechanical challenges.

## 5. Conclusions

The present in vitro study demonstrated that Beautifil Bulk Restorative exhibited the highest surface microhardness values both before and after thermocycling, followed by Fuji II LC and EQUIA Forte HT. All tested materials showed a statistically significant reduction in microhardness after thermal cycling, highlighting the negative impact of simulated aging on their surface mechanical properties. These findings indicate that material composition plays a crucial role in resistance to thermal aging in terms of surface hardness. However, as the present study evaluated only surface microhardness under controlled laboratory conditions, the results should be interpreted as indicative of mechanical behavior rather than definitive clinical performance. Further investigations incorporating additional mechanical parameters and clinical data are required to clarify the implications of these findings for restorative material selection in pediatric dentistry.

## Figures and Tables

**Table 1:** The composition and manufacturers of the materials tested in this study.

Material	Composition	Manufacturer
Beautifil Bulk Restorative	Giomer-based, containing Bis-GMA, UDMA, Bis-MPEPP, TEGDMA, and surface pre-reacted glass (S PRG) fillers; includes polymerization initiators, pigments, and additives	SHOFU Inc., Kyoto, Japan
EQUIA Forte HT	High-viscosity glass ionomer reinforced with fluoroaluminosilicate glass, polyacrylic acid, iron oxide, polybasic carboxylic acid, and water	GC Co.,Tokyo, Japan
Fuji II LC	Resin-modified GIC containing HEMA, TEGDMA, polyacrylic acid, and fluoroaluminosilicate glass	GC Co., Tokyo, Japan

**Table 2:** Vickers Microhardness Values (Mean ± SD) of Restorative Materials Before and After Thermal Cycling. (n = 15 for each subgroup).

Material	Condition	Mean ± SD	*p* Value
Beautifil Bulk Restorative Group 1	Before thermal cycling Group 1a	218.69 ± 3.77	*p*< 0.001
	After thermal cycling Group 1b	180.86 ± 3.37	
EQUIA Forte HT Group 2	Before thermal cycling Group 2a	87.14 ± 2.22	*p* < 0.001
	After thermal cycling Group 2b	65.08 ± 2.15	
Fuji II LC Group 3	Before thermal cycling Group 3a	129.66 ± 1.68	*p*< 0.001
	After thermal cycling Group 3b	89.27 ± 2.65	

Intragroup comparisons were performed using the Wilcoxon signed-rank test, and intergroup comparisons were conducted using the Kruskal–Wallis H test followed by Mann–Whitney U test.

## Data Availability

The datasets generated during the current study are available from the corresponding author upon reasonable request. The data are not publicly available due to institutional policies regarding data sharing.
